# Supplementary material for: Jak2 and Jaw Muscles Are Required for Buccopharyngeal Membrane Perforation during Mouth Development
Source: J Dev Biol. 2023 May 31;11(2):24. doi: 10.3390/jdb11020024 (PMC10298892; doi:10.3390/jdb11020024)
Supplement: Supplementary file 1 [file jdb-11-00024-s001.zip › jdb-2367752-supplementary figures.pdf]

Supplementary Materials

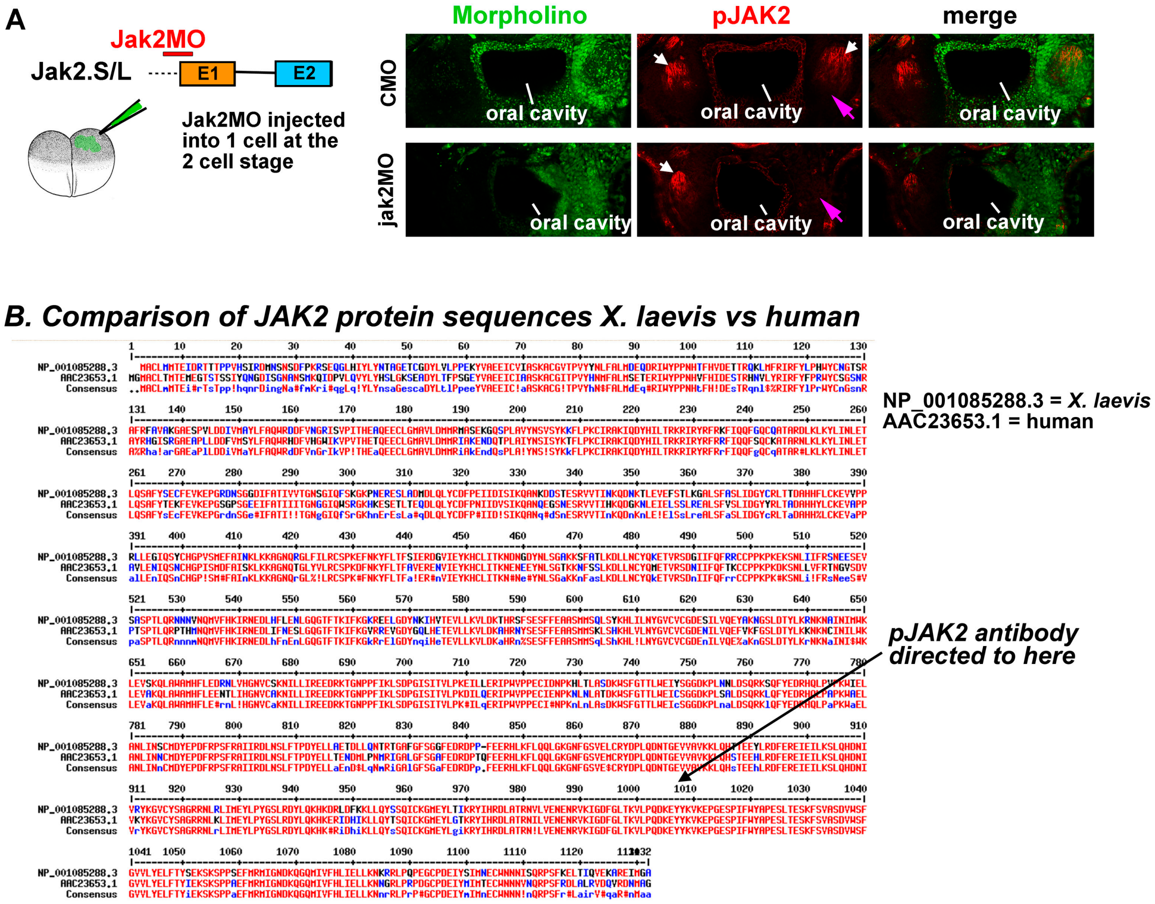

Figure S1. Jak2 protein sequence and validation of the morpholino.

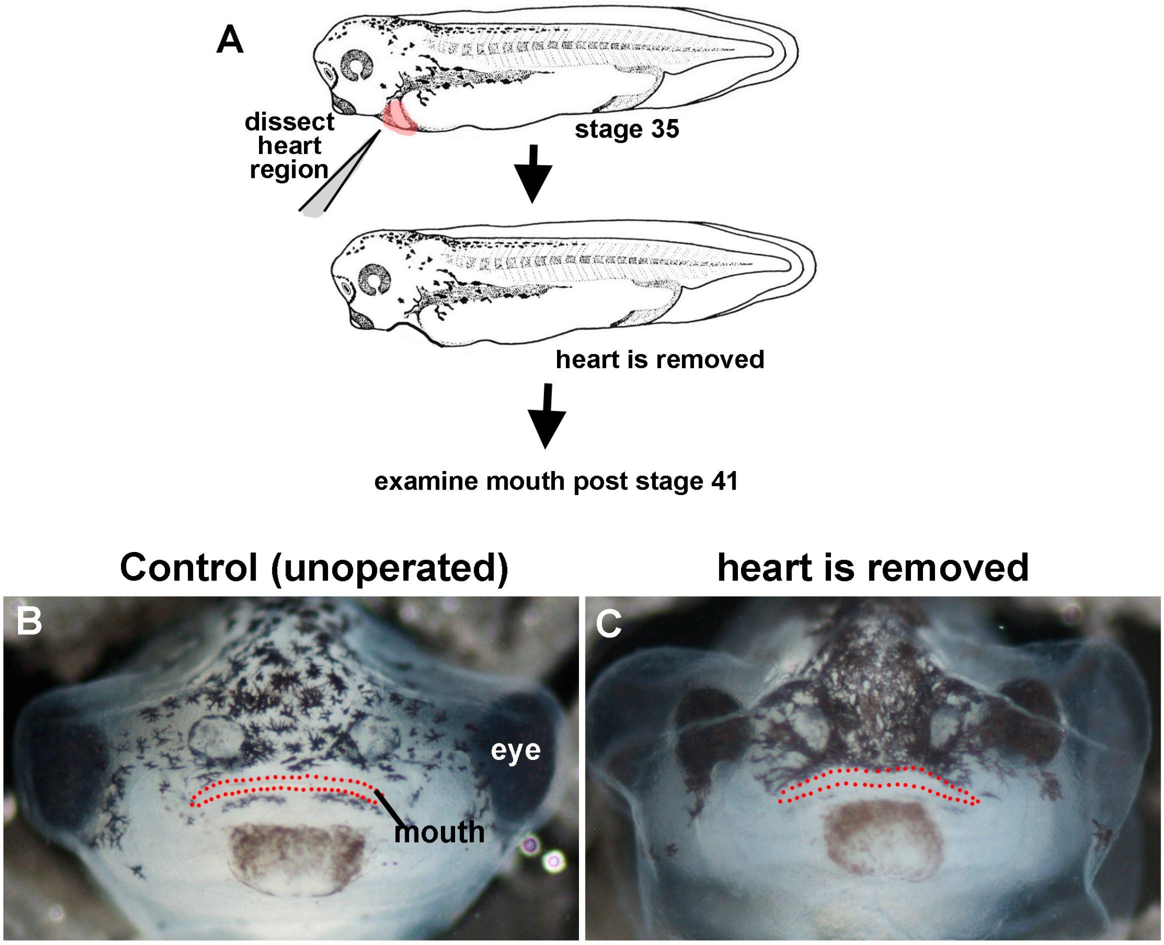

Figure S2. Removing the heart has no effect on buccopharyngeal membrane rupture and Cytochalasin D treatment has no effect on muscle development.

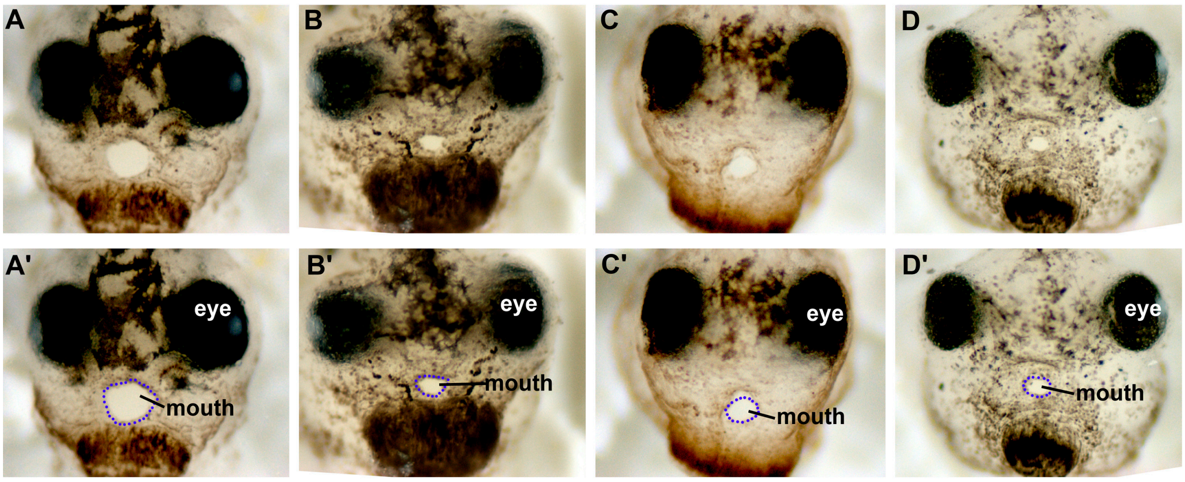

Figure S3. Face shape and size does not correlate with buccopharyngeal membrane perforation.

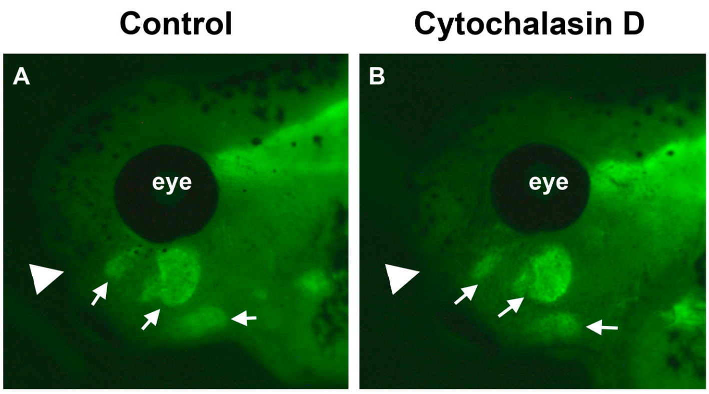

Figure S4. Cytochalasin D treatment does not overtly affect muscle development.
